# Association between SII and postoperative pulmonary infection in elderly patients undergoing laparoscopic abdominal surgery

**DOI:** 10.3389/fmed.2025.1532040

**Published:** 2025-04-04

**Authors:** Qiuping Wen, Zhenming Kang, Zhiyong Shen

**Affiliations:** ^1^Department of Anesthesiology, Quanzhou First Hospital Affiliated to Fujian Medical University, Quanzhou, China; ^2^Department of Respiratory and Critical Care Medicine, Jinjiang Municipal Hospital, Quanzhou, China

**Keywords:** postoperative pulmonary infections, laparoscopic abdominal surgery, elderly patients, systemic immune-inflammation index (SII), risk factors

## Abstract

**Objectives:**

This study aimed to identify the contributing factors, including systemic immune-inflammation index (SII), for pulmonary infections in elderly patients undergoing laparoscopic abdominal surgery with tracheal intubation under general anesthesia.

**Methods:**

A total of 356 eligible elderly patients were monitored post-surgery for pneumonia. Pathogens were identified from sputum samples, and factors like age, smoking, chronic obstructive pulmonary disease (COPD), intubation, anesthesia duration, and SII were analyzed for their influence on infection risk.

**Results:**

The pathogens were predominantly Gram-negative bacteria, with *Klebsiella pneumoniae* being the most common (26%). Significant risk factors for developing pneumonia included advanced age, smoking, COPD, prolonged intubation, and extended anesthesia. A higher preoperative SII was associated with an increased risk of pneumonia and correlated with infection severity and elevated levels of inflammatory markers. Multivariate analysis identified age over 70 (OR = 1.273, *p* = 0.021), age over 80 (OR = 2.085, *p* < 0.001), COPD (OR = 1.528, *p* = 0.009), prolonged intubation >2 h (OR = 2.187, *p* < 0.001), general anesthesia >4 h (OR = 1.846, *p* = 0.006), operative time > 2 h (OR = 1.415, *p* = 0.016), and preoperative SII >489.3 (OR = 1.403, *p* = 0.012) as independent risk factors for postoperative pulmonary infection.

**Conclusion:**

Our study highlighted critical risk factors for postoperative pulmonary infections in elderly patients undergoing laparoscopic abdominal surgery. Preoperative SII could be a novel predictor, offering potential for improved pre-surgical risk assessment.

## Introduction

Postoperative pulmonary infections (PPIs) are a significant complication in surgical patients, particularly those undergoing laparoscopic abdominal surgeries involving tracheal intubation and general anesthesia (1–4). Tracheal intubation, while essential for airway management, has been identified as a contributing factor to increased PPI incidence, with prevalence rates of lower respiratory tract infections post-intubation ranging from 2.8 to 24.7% (5, 6). The disruption of upper respiratory tract barrier functions and inhibition of ciliary activity in the trachea during general anesthesia elevate infection risks (7, 8). With hospital-acquired pneumonia rates as high as 10.7% following abdominal surgeries (9), PPIs have become a crucial determinant of patient morbidity and mortality (10), substantially impacting the duration of hospital stays and overall patient survival.

The aging global population presents additional challenges, as older patients are more susceptible to respiratory infections post-surgery due to decreased respiratory and immune functions (2, 11). The decreased respiratory and immune functions in the elderly further amplify the risks associated with tracheal intubation and general anesthesia, leading to a higher propensity for PPI and subsequent respiratory failure or multiorgan dysfunction. As a result, timely and effective prevention and control of PPI are paramount to reducing mortality rates in this patient population.

The systemic immune-inflammation index (SII), a biomarker of systemic inflammatory response readily calculated from three complete blood count parameters, has emerged as a significant prognostic tool in various malignancies (12, 13). This study was dedicated to evaluating the prognostic value of the preoperative SII in predicting PPI and its correlation with infection severity in elderly patients undergoing laparoscopic abdominal surgeries with tracheal intubation and general anesthesia. Through this investigation, we aimed to elucidate the potential of SII as a predictive marker, thereby facilitating improved clinical outcomes and guiding preventive strategies against PPI.

## Methods

### Patient selection and study design

This study was approved by the ethics committee of Quanzhou First Hospital Affiliated to Fujian Medical University. Patients were recruited based on the following inclusion criteria: elderly patients (≥60 years old) undergoing elective laparoscopic abdominal surgery at our institution; American Society of Anesthesiologists (ASA) physical status classification I-III; surgery types encompassing gastrointestinal, hepatobiliary, spleen and pancreas, and urinary system procedures; and availability of complete clinical data.

### Exclusion criteria

Patients diagnosed with pneumonia prior to anesthesia, those with recent history of cardiopulmonary resuscitation or artificial airway establishment, patients with concurrent immunodeficiency, hematological, oncological disorders, severe cardiopulmonary or renal diseases, or unstable vital signs were excluded from the study.

### Diagnosis criteria for pulmonary infection

Patients were diagnosed with hospital-acquired pneumonia based on the following criteria: (1) new onset of productive cough with purulent sputum, with or without chest pain; (2) fever (body temperature > 37.5°C); (3) physical examination findings of lung consolidation and/or moist rales; (4) peripheral blood white blood cell count >10 × 10^9^/L or <4 × 10^9^/L, with or without a left shift; (5) imaging studies showing new infiltrates, consolidation, or interstitial patterns on chest X-ray or CT scan, with or without pleural effusion. A diagnosis of pulmonary infection was established after excluding lung cancer, pulmonary edema, aspiration, foreign body inhalation, tuberculosis, pulmonary embolism, and non-infectious interstitial lung diseases, based on at least one of the first four criteria in conjunction with the fifth criterion.

Throughout their hospital stay, patients were closely monitored for signs of pulmonary infection. Daily clinical assessments included vital sign measurements every 4 h, chest auscultation twice daily, daily evaluation of sputum characteristics, and continuous oxygen saturation monitoring. Chest X-rays were performed on postoperative day 1 and as clinically indicated thereafter. Blood tests, including complete blood count and C-reactive protein, were conducted on postoperative days 1, 3, and 5, and as needed based on clinical suspicion of infection.

### Anesthesia management

Elderly patients underwent general anesthesia with endotracheal intubation. Upon entering the operating room, an upper limb vein was cannulated, and sodium lactate Ringer’s solution was infused. Standard monitoring including electrocardiography (ECG), pulse oximetry (SpO_2_), non-invasive blood pressure (MAP), heart rate (HR), end-tidal carbon dioxide pressure (PETCO_2_), and bispectral index (BIS) was established. Anesthesia was induced with intravenous administration of midazolam (0.04 mg/kg), remifentanil (0.05–1 μg/kg), propofol (1–2 mg/kg), and cisatracurium (0.1–0.15 mg/kg). Upon achieving adequate muscle relaxation, endotracheal intubation was performed followed by controlled ventilation. Tidal volume was set at 6–8 mL/kg, with an I:E ratio of 1:2, and respiratory rate adjusted to 12–15 breaths/min. Anesthesia was maintained with adjustments based on blood pressure, heart rate, and surgical stimulus. MAP and HR were maintained within ±20% of preoperative values, BIS between 40 and 60, and PETCO2 between 35 and 45 mmHg. Acid–base and electrolyte imbalances were corrected based on arterial blood gas analysis. Cisatracurium was discontinued 30 min before the end of surgery, and the tracheal tube was removed when the patient regained consciousness, spontaneous breathing, strong cough, and swallowing reflexes, and SpO2 remained above 95%.

### Collection of sputum samples

Sputum samples were collected exclusively from patients diagnosed with pulmonary infection, rather than from all extubated patients. For these cases, sterile suction catheters were used to obtain respiratory secretions *via* artificial airways. Specimens were deemed valid for culture only if microscopic examination revealed >25 leukocytes and <10 squamous epithelial cells per high-power field, a standardized approach that minimizes contamination risk. Bacterial identification was conducted using an automated system (ATB Expression, bioMérieux), with quality control strains sourced from the China General Microbiological Culture Collection Center.

### Blood sample collection and SII calculation

Blood samples were collected after at least 8 h of fasting within 24 h before the surgery. The preoperative SII was calculated as follows: SII = neutrophil count (10^9^/L) × platelet count (10^9^/L)/lymphocyte count (10^9^/L).

## Results

**Figure 1:**
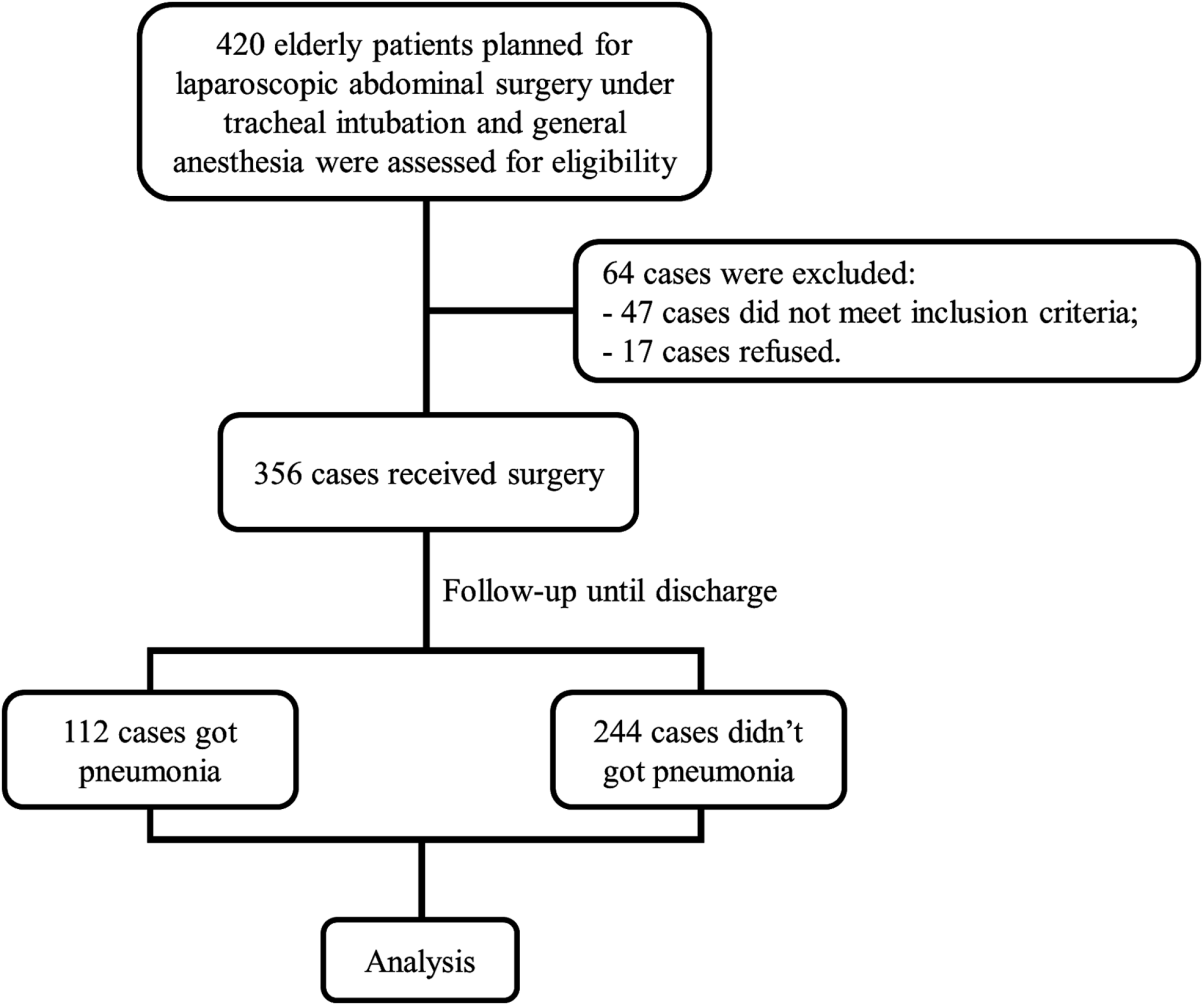
Patient enrollment and outcomes flowchart. The flowchart depicting the progression of the study. Out of 420 assessed elderly patients, 356 were eligible and underwent laparoscopic abdominal surgery. Post-surgery, 112 patients developed pneumonia, while 244 did not. These cases were then advanced to the analysis phase.

In this study, a cohort of 420 elderly patients planned for laparoscopic abdominal surgery under general anesthesia were assessed for eligibility (Figure 1). Of these, 356 met the inclusion criteria and consented to participate. Throughout the hospitalization period following surgery, 112 cases were identified with varying degrees of pneumonia, while 244 cases did not develop this complication. The flow of patient selection and follow-up is depicted in Figure 1, which outlines the process from initial assessment through to the analysis stage, accounting for exclusions due to failure to meet inclusion criteria, refusal to participate, and the incidence of postoperative pneumonia.

### Distribution of pathogens in postoperative pulmonary infections

After the surgical procedures, a subset of 112 patients developed pulmonary infections, as opposed to 244 patients who remained infection-free (Table 1). For the diagnosis of pulmonary infections, respiratory tract sputum samples were obtained via a sterile disposable suction catheter and analyzed. Samples with white blood cell counts >25/HP field and squamous cells <10/HP field were cultured for pathogen isolation and identification using the ATB Expression automatic bacterial identification system from bioMérieux, France. Quality control strains were acquired from the China General Microbiological Culture Collection Center. A total of 139 pathogenic bacteria were cultured, with the distribution of pathogens shown in Table 1. Notably, Gram-negative bacteria were predominant, with *Klebsiella pneumoniae* being the most common, accounting for 26.6% of the isolates, followed by *Pseudomonas aeruginosa* and *Acinetobacter baumannii*, constituting 18.0 and 16.5%, respectively. Gram-positive bacteria and fungi were also present but in smaller proportions.

**Table 1:** Pathogenic bacteria distribution of postoperative pulmonary infection in elderly patients received laparoscopic abdominal surgery under tracheal intubation and general anesthesia.

Pathogenic bacteria distribution	*n* = 139	Ratio (%)
Gram-positive bacteria		
*Staphylococcus aureus*	13	9.4
*Staphylococcus epidermidis*	10	7.2
*Streptococcus hemolyticus*	7	5.0
Gram-negative bacteria		
*Pseudomonas aeruginosa*	25	18.0
*Klebsiella pneumoniae*	37	26.6
*Acinetobacter baumannii*	23	16.5
*Escherichia coli*	12	8.6
*Enterobacter cloacae*	4	2.9
Fungus	8	5.8

### Age and perioperative factors associated with postoperative pulmonary infection

The analysis of baseline and perioperative characteristics in elderly patients undergoing laparoscopic abdominal surgery reveals significant disparities between those who developed postoperative pulmonary infection and those who did not. A stark contrast is observed in age distribution, with only 17.0% of patients aged 60–69 developing pneumonia versus 33.9% in the ≥80 age group (*p* < 0.001). Smoking habits and the presence of chronic obstructive pulmonary disease (COPD) also showed a significant association with postoperative infections; 34.8% of smokers experienced pneumonia compared to 21.7% of non-smokers (*p* = 0.013), and COPD patients had a higher incidence (17.9%) than those without (7.4%, *p* = 0.005). Moreover, prolonged tracheal intubation (≥2 h) and general anesthesia (≥4 h) were significantly linked to higher rates of pneumonia (*p* < 0.001 for both). These findings underscore the importance of considering these factors as potential risk enhancers for pulmonary complications post-surgery. The data suggest that elderly patients, especially those over 80, smokers, and those with COPD or requiring longer anesthesia and intubation, should be closely monitored for the development of pulmonary infection following laparoscopic surgery.

### Preoperative SII as a predictor of postoperative pulmonary infection

**Figure 2:**
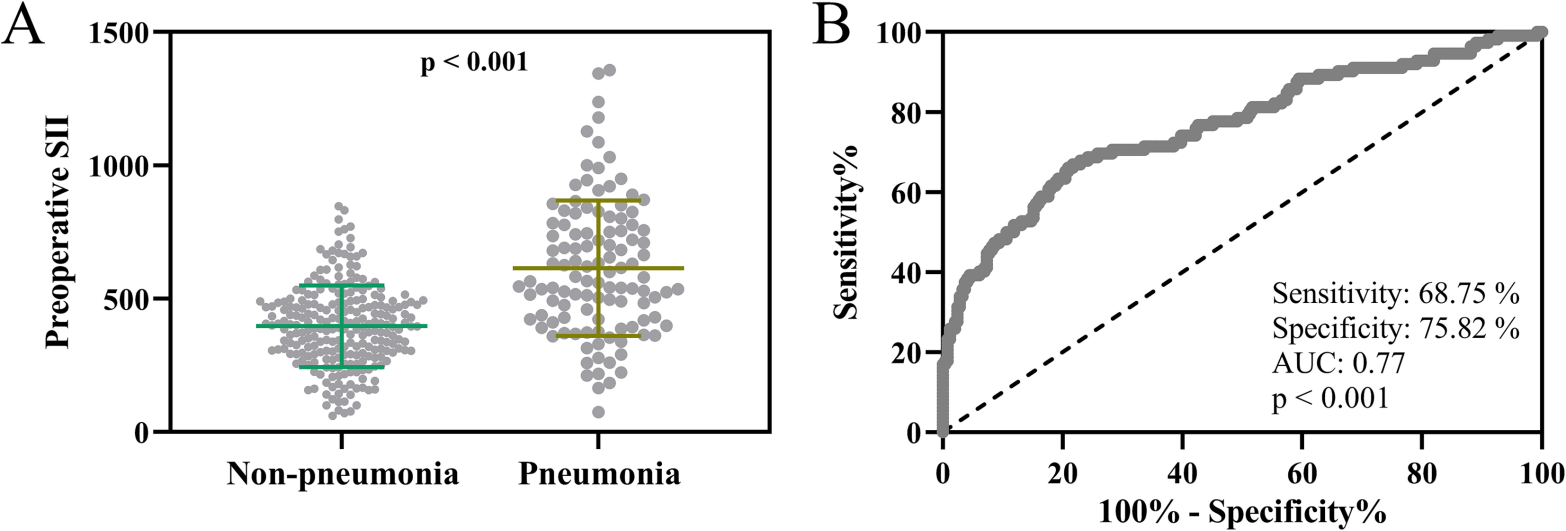
Preoperative systemic immune-inflammation index as a predictor of postoperative pulmonary infection in elderly laparoscopic surgery patients. **(A)** Comparison of preoperative systemic immune-inflammation index (SII) between elderly patients with (*n* = 112) and without (*n* = 244) postoperative pulmonary infection who received laparoscopic abdominal surgery under tracheal intubation and general anesthesia. Data points represent individual patients with overlaying mean ± standard deviation (SD), and statistical significance assessed using an unpaired t-test with Welch’s correction (*p* < 0.001). **(B)** Receiver operating characteristic (ROC) curve evaluating the prognostic performance of preoperative SII in predicting postoperative pulmonary infection among elderly patients undergoing laparoscopic abdominal surgery. The area under the curve (AUC), sensitivity, and specificity are indicated, with statistical significance noted (*p* < 0.001).

The comparative analysis of preoperative SII underscored its potential as an indicator for postoperative pulmonary infection risk. The mean preoperative SII was notably higher in patients who later developed pneumonia (*n* = 112) compared to those who did not (*n* = 244), an association that proved statistically significant (*p* < 0.001, Figure 2A). This elevated preoperative immune-inflammatory response might be a harbinger of increased susceptibility to pulmonary complications after surgery. Additionally, the ROC curve analysis indicated that preoperative SII has a reliable predictive value for postoperative pulmonary infection, offering a sensitivity of 68.75% and specificity of 75.82% (AUC = 0.77, *p* < 0.001, Figure 2B). This predictive capacity positions preoperative SII as a valuable tool for identifying at-risk elderly patients undergoing laparoscopic surgery under general anesthesia with tracheal intubation.

### Independent risk factors for postoperative pulmonary infection

To confirm the independent risk factors for postoperative pulmonary infection in elderly patients who underwent laparoscopic abdominal surgery with tracheal intubation and general anesthesia, we performed the multivariate logistic regression analysis, building upon the univariate analysis from Table 2 and the preoperative SII comparisons. We found that patients aged over 70 and 80 had increased odds of infection, with odds ratios (OR) of 1.273 and 2.085, respectively, indicating a higher risk as age advances (*p* = 0.021 and *p* < 0.001, Table 3). The presence of COPD also increased the risk (OR = 1.528, *p* = 0.009). Furthermore, prolonged tracheal intubation over 2 h (OR = 2.187, *p* < 0.001) and general anesthesia lasting more than 4 h (OR = 1.846, *p* = 0.006) were associated with a heightened risk of pulmonary infection. An operative time exceeding 2 h (OR = 1.415, *p* = 0.016) and a preoperative SII greater than 489.3 (OR = 1.403, *p* = 0.012) were also identified as significant independent risk factors. These findings assert the importance of these variables as predictors for developing pulmonary infection during the postoperative hospital stay in elderly patients undergoing such surgeries.

**Table 2:** Baseline and perioperative characteristics of elderly patients with and without postoperative pulmonary infection who received laparoscopic abdominal surgery under tracheal intubation and general anesthesia.

Characteristics	Non-pneumonia (*n* = 244)	Pneumonia (*n* = 112)	*p*-value
Age (years)			
60–69	99 (40.6%)	19 (17.0%)	<0.001
70–79	104 (42.6%)	55 (49.1%)	
≥80	41 (16.8%)	38 (33.9%)	
Gender			
Male	128 (52.5%)	51 (45.5%)	0.254
Female	116 (47.5%)	61 (54.5%)	
Type of surgery			
Gastrointestinal	97 (39.8%)	43 (38.4%)	0.699
Hepatobiliary	89 (36.5%)	47 (42.0%)	
Spleen and pancreas	23 (9.4%)	10 (8.9%)	
Urinary system	35 (14.3%)	12 (10.7%)	
COPD			
Yes	18 (7.4%)	20 (17.9%)	0.005
No	226 (92.6%)	92 (82.1%)	
Smoking			
Yes	53 (21.7%)	39 (34.8%)	0.013
No	191 (78.3%)	73 (65.2%)	
Hypertension			
Yes	92 (37.7%)	37 (33.0%)	0.409
No	152 (62.3%)	75 (67%)	
Diabetes mellitus			
Yes	56 (22.9%)	34 (30.4%)	0.149
No	188 (77.1%)	78 (69.6%)	
Coronary heart disease			
Yes	60 (24.6%)	32 (28.6%)	0.436
No	184 (75.4%)	80 (71.4%)	
Tracheal intubation duration (hours)			
<2 h	155 (63.5%)	44 (39.3%)	<0.001
≥2 h	89 (36.5%)	68 (60.7%)	
General anesthesia duration (hours)			
<4 h	134 (54.9%)	37 (33.1%)	<0.001
≥4 h	110 (45.1%)	75 (66.9%)	
Operation time (hours)			
<2 h	137 (56.1%)	46 (41.1%)	0.009
≥2 h	107 (43.9%)	66 (58.9%)	

The data were shown as n (percentage). The comparisons of data between the two groups were done by Fisher’s exact test or Chi-square test.

COPD, chronic obstructive pulmonary disease.

**Table 3:** Multivariate logistic analysis for the postoperative pulmonary infection in elderly patients received laparoscopic abdominal surgery under tracheal intubation and general anesthesia.

	OR	95% CI	*p*-value
Age more than 70	1.273	1.091 to 3.114	0.021
Age more than 80	2.085	1.429 to 3.972	< 0.001
Smoking	1.372	0.926 to 2.331	0.094
Complicated COPD	1.528	1.134 to 2.746	0.009
Tracheal intubation duration more than 2 h	2.187	1.332 to 4.249	< 0.001
General anesthesia duration more than 4 h	1.846	1.264 to 4.674	0.006
Operation time more than 2 h	1.415	1.073 to 2.835	0.016
Preoperative SII more than 489.3	1.403	1.058 to 3.275	0.012

OR, odds ratio; CI, confidence interval; COPD, chronic obstructive pulmonary disease; SII, systemic immune-inflammation index.

### Correlation of inflammatory markers with preoperative SII

**Figure 3:**
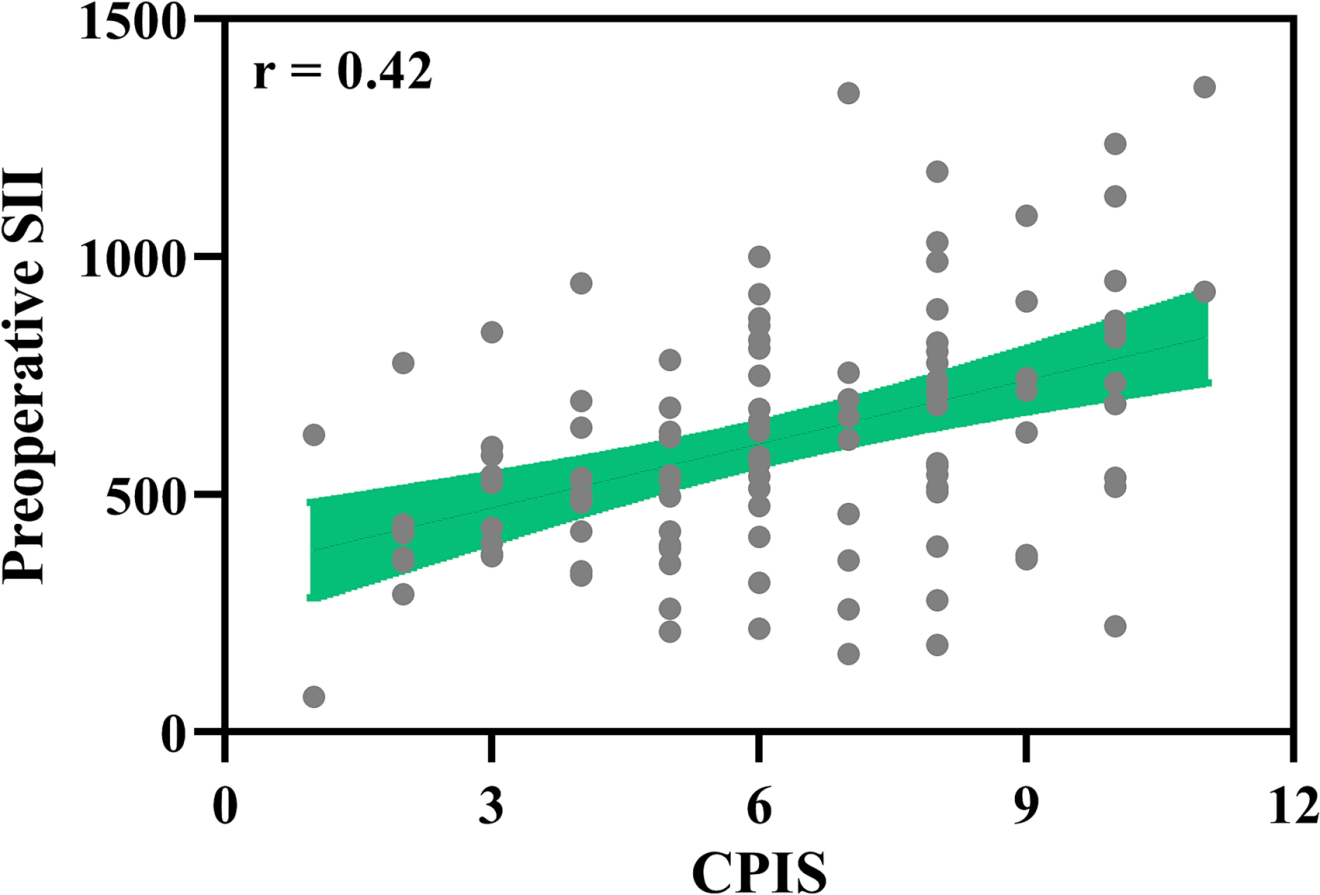
Correlation of clinical pulmonary infection score (CPIS) with preoperative SII. Spearman correlation analysis of clinical pulmonary infection score (CPIS) at diagnosis of pulmonary infection with preoperative systemic immune-inflammation index (SII) in elderly patients with postoperative pulmonary infection who received laparoscopic abdominal surgery under tracheal intubation and general anesthesia (*n* = 112). *p* < 0.001.

To investigate whether there are correlations between preoperative systemic immune inflammation and infection severity, we analyzed the d spearman correlation between the Clinical Pulmonary Infection Score (CPIS) at the time of pulmonary infection diagnosis and the preoperative SII in elderly patients who underwent laparoscopic abdominal surgery with tracheal intubation and general anesthesia (*n* = 112, Figure 3). The CPIS, with scores ranging up to 12, is a composite measure that includes clinical, laboratory, imaging, and microbiological criteria to evaluate the severity of suspected pneumonia. The analysis revealed a positive correlation (*r* = 0.42, *p* < 0.001), indicating that patients with higher preoperative SII tend to have more severe infections postoperatively, necessitating more aggressive anti-infection interventions.

**Figure 4:**
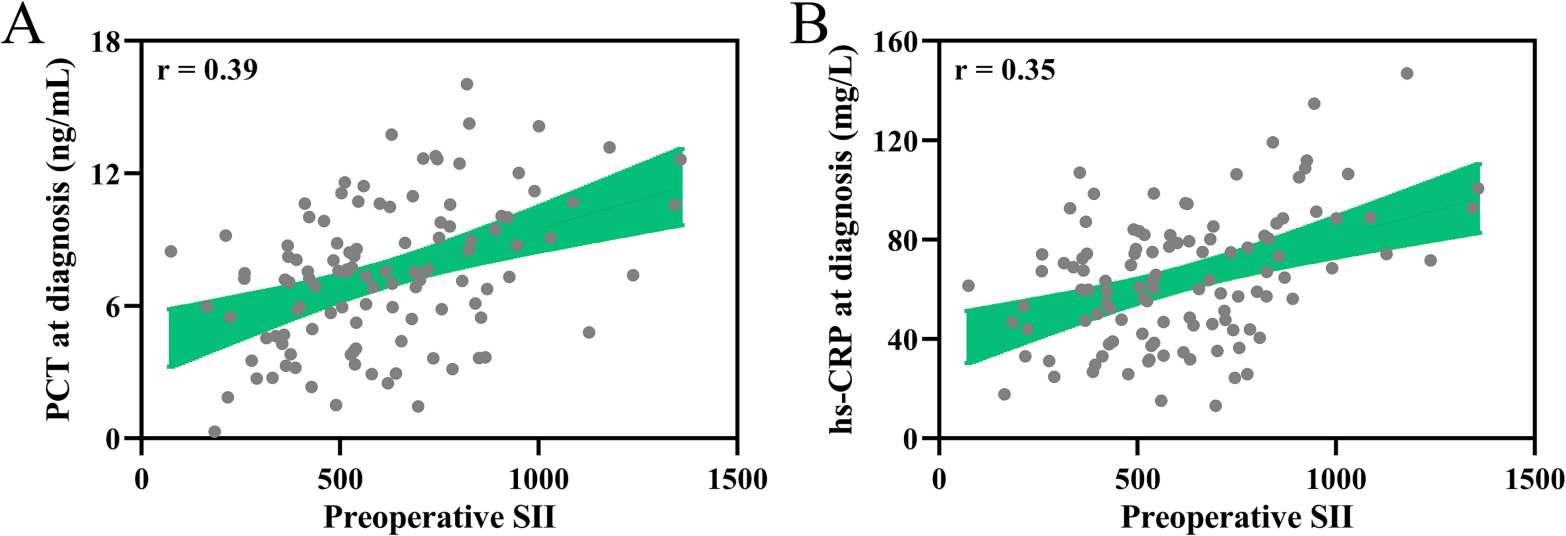
Association between preoperative systemic immune-inflammation index (SII) and severity of infection in patients. Spearman correlation analysis of preoperative systemic immune-inflammation index (SII) with the concentrations of PCT in plasma **(A)**, serum hs-CRP **(B)** at diagnosis of pulmonary infection in elderly patients with postoperative pulmonary infection who received laparoscopic abdominal surgery under tracheal intubation and general anesthesia (*n* = 112). *p* < 0.001.

Additionally, the correlation analysis between the preoperative SII and the levels of plasma procalcitonin (PCT) and serum high-sensitivity C-reactive protein (hs-CRP) measured at the time of pulmonary infection diagnosis in the same cohort of patients (Figures 4A,B). Both inflammatory markers showed a significant positive correlation with the preoperative SII (*p* < 0.001 for both), demonstrating that a higher SII is associated with increased concentrations of PCT and hs-CRP during infection. These findings support the preoperative SII as a robust indicator of inflammation and potential predictor of infection severity in elderly patient post-surgery.

## Discussion

With the worldwide trend of aging populations and extended life spans, the surgical needs of the elderly are increasingly prominent. The rise in laparoscopic surgery could be attributed to enhanced surgical training, augmented expertise in the field, and evidence of superior patient outcomes. Laparoscopy offers significant benefits, including diminished intraoperative bleeding, reduced postoperative discomfort, decreased duration of hospitalization, expedited patient mobilization, quicker resumption of routine activities, and a reduced risk of pulmonary, thrombotic, and abdominal wall complications. As a result, laparoscopy has become the preferred surgical approach for a variety of prevalent medical conditions (14). However, postoperative pulmonary infection frequently occurs following laparoscopic surgery, making it essential to implement evidence-based nursing care during the perioperative phase to aid in patient prognosis and recovery (15). Crucially, identifying pre-anesthesia risk factors associated with postoperative pulmonary infections in elderly patients who have undergone laparoscopic abdominal surgery could markedly bolster the recuperation process in this demographic. In this study, we meticulously examined the correlation between various factors such as age, smoking status, the presence of COPD, duration of tracheal intubation, and SII, and the elevated risk of postoperative pulmonary infections in elderly patients undergoing laparoscopic abdominal surgery. These results not only corroborate previously established risk factors but also introduce new variables, like preoperative SII, into the risk management paradigm for elderly patients facing such procedures.

Laparoscopic methods are widely used in the aging population. Presently, over 40% of laparoscopic surgeries in the U.S. are conducted on individuals above 65 years (16, 17). Laparoscopy offers benefits like fewer wound issues, less post-operative ileus, reduced intraoperative bleeding, and a diminished need for post-surgical rehabilitation, and is considered safe and beneficial for the elderly (18, 19). Nonetheless, laparoscopic surgery is not without its risks, particularly for the elderly who generally have higher rates of surgical morbidity and mortality. Prior studies have highlighted that despite the smaller incisions and lower intraoperative hemorrhage of laparoscopic surgeries, they could still lead to considerable physiological stress caused by peritoneal insufflation and elevated intra-abdominal pressure (20), which may result in an increased release of proinflammatory cytokines and acute-phase proteins, potentially heightening the risk of postoperative infections (21). We found that the notable incidence of postoperative pneumonia in our study (31.7%) is significantly higher than what has been reported in certain previous studies (15, 22, 23), particularly those involving younger cohorts or different types of surgeries. The most frequently found pathogenic bacteria was *Klebsiella pneumoniae*. This discrepancy underscores the unique vulnerability of the elderly population, a demographic that is expanding globally and increasingly undergoing surgical interventions. The observed predominance of Gram-negative bacteria, especially *Klebsiella pneumoniae*, as the causative pathogens for postoperative infections in our study, echoes the findings of recent literature indicating a shift in the microbial landscape of hospital-acquired infections. An analysis identified risk factors for pulmonary infection included hypoproteinemia, diabetes, a history of pulmonary diseases, and blood transfusions during the perioperative period and the primary causative pathogens were found to be *Klebsiella pneumoniae*, *Staphylococcus aureus*, *Pseudomonas aeruginosa*, *Escherichia coli*, and *Streptococcus pneumoniae* (15). This has substantial implications for antibiotic stewardship and infection control practices in the perioperative care of elderly patients.

Our study found the correlation of increased age with a higher risk of postoperative pneumonia aligns with the previous research (24). For instance, studies have consistently shown that advanced age is a significant risk factor for postoperative complications, primarily due to decreased physiological reserves and the presence of comorbid conditions more common in this demographic (25). The significant association between smoking, COPD, and the increased incidence of postoperative pneumonia found in our study resonates with findings from previous research (25–27). Smoking and COPD are well-documented risk factors for respiratory complications, given their detrimental impact on pulmonary function. This necessitates a more vigilant approach to perioperative management in patients with these risk factors. In addition, our emphasis on prolonged anesthesia and tracheal intubation as risk factors for postoperative pulmonary infection is a crucial addition to existing knowledge.

The SII integrates neutrophil, lymphocyte, and platelet counts to assess both local immune responses and systemic inflammation, and has been linked to poor outcomes in various diseases such as coronary heart disease (28). SII is frequently utilized to evaluate systemic inflammation and immune response, offering a comprehensive assessment by incorporating neutrophils, lymphocytes, and platelets. While other indicators such as neutrophil-to-lymphocyte ratio (NLR) and platelet-to-lymphocyte ratio (PLR) are also employed clinically, they provide insights into different aspects of physiological status. NLR primarily reflects immune dysregulation and serves as a prognostic marker for disease severity and mortality, focusing on the balance between innate and adaptive immune responses. PLR, representing the relationship between platelet and lymphocyte levels, has emerged as an indicator of inflammation, platelet activation, and atherosclerosis, with particular relevance to cardiovascular health. When compared to NLR and PLR, SII has shown superior predictive accuracy in several clinical scenarios. For instance, in lung cancer surgery, a higher preoperative SII has been identified as an independent risk factor for postoperative pulmonary infections. Notably, SII demonstrated greater predictive accuracy for both the incidence and severity of these infections compared to preoperative NLR and PLR (29). This finding underscores the potential of SII as a more reliable predictor in perioperative risk assessment. Recent studies have also highlighted the strong correlation between SII and infectious diseases. High SII levels have been independently associated with increased in-hospital mortality in conditions such as infective endocarditis (30, 31). This association further emphasizes the versatility of SII as a prognostic tool across different medical disciplines.

In our study, we identified the SII as a valuable pre-anesthesia risk assessment tool. This predictor emerged as a significant indicator of postoperative pulmonary infection risk, offering potential for improved pre-surgical risk stratification in elderly patients undergoing laparoscopic abdominal surgery. This aligns with the current understanding of the role of systemic inflammation in postoperative outcomes. Additionally, we observed a significant difference in NLR between elderly patients with (*n* = 112) and without (*n* = 244) postoperative pulmonary infection who underwent laparoscopic abdominal surgery under tracheal intubation and general anesthesia (Supplementary Figure S1). However, when comparing predictive performance using ROC analysis, we found that the AUC for NLR was 0.69, which is lower than the AUC of 0.77 for SII. This indicates that SII demonstrates superior predictive efficacy compared to NLR in our study population. While NLR is indeed a valuable indicator, our results suggest that SII, which incorporates neutrophil, lymphocyte, and platelet counts, may provide a more comprehensive assessment of the inflammatory status and thus serve as a better predictor of postoperative pulmonary infection in this specific patient group. Furthermore, our study suggests that certain perioperative management strategies, such as the duration of anesthesia and tracheal intubation, should be carefully considered in the elderly. These factors, if not optimally managed, could significantly increase the risk of postoperative pulmonary infections, as evidenced by our findings, and supported by existing literature (2).

While our study sheds light on these crucial aspects, it is not without limitations. The specificity of our study to elderly patients undergoing laparoscopic abdominal surgery means that our findings might not be directly applicable to other surgical populations or procedures. The observational nature of our study precludes establishing causality, and our findings are based on associations. Additionally, although abdominal infections are common following abdominal surgeries, our study specifically focused on pulmonary infections, which may not capture the full spectrum of postoperative complications. Furthermore, due to insufficient postoperative blood test data for SII within 8–24 h after surgery, we were unable to assess the predictive value of early postoperative SII for pulmonary infections, which could have provided additional insights. These limitations highlight areas for future research and underscore the need for cautious interpretation of our results within the context of our specific study population and design.

## Conclusion

In conclusion, our study identifies key risk factors for postoperative pulmonary infections in elderly patients undergoing laparoscopic abdominal surgery with tracheal intubation under general anesthesia. We found that advanced age, smoking history, COPD, prolonged tracheal intubation, extended general anesthesia, and elevated preoperative SII are significant predictors of postoperative pulmonary infections. Notably, the preoperative SII emerged as a novel and valuable predictor, demonstrating potential as a pre-anesthesia risk assessment tool. These findings emphasize the importance of comprehensive pre-anesthesia risk evaluation and tailored perioperative management strategies in this vulnerable patient population.

## Data Availability

The original contributions presented in the study are included in the article/Supplementary material, further inquiries can be directed to the corresponding authors.
