# Supplementary material for: Association between SII and postoperative pulmonary infection in elderly patients undergoing laparoscopic abdominal surgery
Source: Front Med (Lausanne). 2025 Apr 4;12:1532040. doi: 10.3389/fmed.2025.1532040 (PMC12006073; doi:10.3389/fmed.2025.1532040)
Supplement: Supplementary file 1 [file Data_Sheet_1.pdf]

Supplementary materials

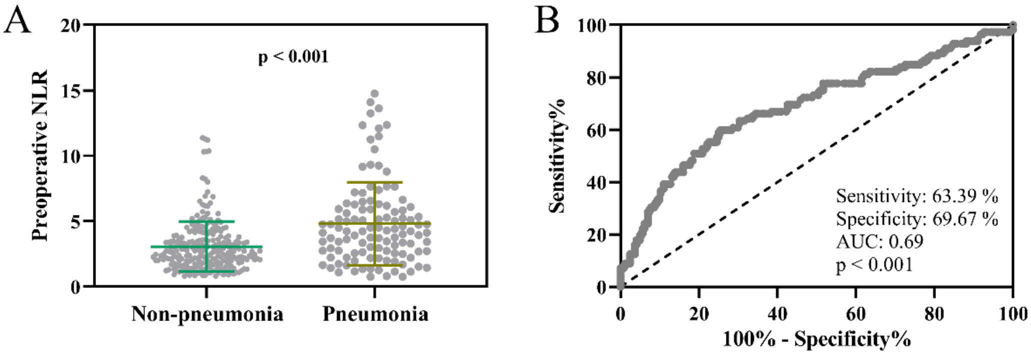

Figure S1. A. Comparison of preoperative NLR between elderly patients with (n = 112) and without (n = 244) postoperative pulmonary infection who received laparoscopic abdominal surgery under tracheal intubation and general anesthesia. Data points represent individual patients with overlaying mean ± standard deviation (SD), and statistical significance assessed using a Mann Whitney test ($p < 0.001$). B. Receiver operating characteristic (ROC) curve evaluating the prognostic performance of preoperative NLR in predicting postoperative pulmonary infection among elderly patients undergoing laparoscopic abdominal surgery. The area under the curve (AUC), sensitivity, and specificity are indicated, with statistical significance noted ($p < 0.001$).
